# Metabolomics analysis of follicular fluid in ovarian endometriosis women receiving progestin-primed ovary stimulation protocol for in vitro fertilization

**DOI:** 10.1038/s41598-023-32797-w

**Published:** 2023-04-07

**Authors:** Haiyan Guo, Qianqian Zhu, Hongyuan Gao, Qifeng Lyu, Weiran Chai, Ling Wu, Bin Li

**Affiliations:** Department of Assisted Reproduction, Shanghai Ninth People’s Hospital, Shanghai Jiaotong University School of Medicine, Center for Specialty Strategy Research of Shanghai Jiao Tong University China Hospital Development Institute, Shanghai, 200011 China

**Keywords:** Cell biology, Endocrine system and metabolic diseases, Metabolic disorders, Reproductive disorders

## Abstract

This study aimed to investigate the metabolite profile and inflammatory state of follicular fluid (FF) in women with stage III–IV ovarian endometriosis (OE) who underwent in vitro fertilization (IVF). A cohort of 20 consecutive patients with OE were recruited and received progestin-primed ovary stimulation (PPOS) protocol (study group), while another 20 OE patients received one-month ultra-long term protocol (control group) for IVF in this prospective, nonrandomized study. FF samples were obtained from dominant follicles during oocyte retrieval, and liquid chromatography-mass spectrometry (LC–MS) was used to investigate the metabolites profile of FF. Results showed that significant increases in the levels of proline, arginine, threonine, and glycine in patients who received PPOS protocol compared to the control group (P < 0.05). A panel of three metabolites (proline, arginine, and threonine) was identified as specific biomarkers of OE patients using PPOS protocol. Additionally, levels of interleukin-1β, regulated on activation, normal T cell expressed and secreted, and tumor necrosis factor-α markedly decreased in women who received PPOS protocol compared to the control group (P < 0.05). In conclusion, PPOS protocol regulates the metabolism of several amino acids in the FF, which may play critical roles in the oocyte development and blastocyst formation, and their specific mechanism should be further elucidated.

## Introduction

Endometriosis is an identifiable cause of infertility and also known as a chronic inflammatory status dependent on estrogen^1,2^. In vitro fertilization (IVF) has been a choice for pregnancy in the infertility women. Even though mild ovarian endometriosis (OE) might have an adverse effect on the fertility by affecting the oocyte and embryo development along with embryo implantation^3,4^. Nevertheless, the precise pathophysiological mechanisms underlying the effects of endometriosis on the infertility remain unclear^5–7^. To date, reproductive specialists have devoted to exploring the best IVF regimen for endometriosis patients. Progestins have been applied in the therapy of endometriosis for many years. The therapeutic efficacy of progestins in endometriosis-associated pelvic pain may be owed to its suppression on the regulated on activation, normal T cell expressed and secreted (RANTES) production and inflammatory responses in the pelvis^8^. Fechner et al. proposed that progestins can modulate the local estrodiol (E2) biosynthesis and suppress the growth of ectopic endometrium in the endometriosis women^9^. Some researchers have covered the usage of medroxyprogesterone acetate (MPA) for controlled ovarian hyperstimulation (COH) during IVF in patients, and it is an alternative for the treatment of stage III-IV endometriosis^10,11^. Studies have been conducted to investigate the follicular fluid (FF), aiming to determinate the biomarkers as well as metabolic pathways related to the oocyte and embryo outcomes^12^. The compositions of FF represent the interchanges between oocyte and its associated microenvironment. Oocyte metabolomics profiling may be helpful to investigate the influence of latest assisted reproduction technologies (ART) on the oocyte outcomes^12^. Functional analysis of oocyte quality at the distinctive oocyte level will provide worthy standpoints into the oocyte developmental ability. There is evidence showing that uterine endometriosis has mitochondrial dysfunction; ovarian endometrioma (OMA) has the imbalance between anaerobic glycolysis and β-oxidation. Both of them may affect the fertility of endometriosis women. Since the composition of follicular fluid has been proved to be related to the oocyte development and post fertilization implantation, these findings may help explain the high infertility rate in these patients^13^. In addition, some studies show that endometriosis is related to abnormal lipid metabolism, which can provide the necessary energy to adapt to the inflammatory response by mobilizing lipid metabolism^14^. On the contrary, non-invasive determination of oocyte metabolism have suggested a relationship between metabolism and embryo developmental ability. Metabolomics profile is influenced by the oocyte meiotic progression, age of a patient, regimen of controlled ovarian stimulation (COS) and disease etiology^12,15^. Currently, little is known about the change of cytokines in the local microenvironment of endometriosis, leading to the less acceptance and awareness of the significance of anti-inflammatory factors in the pathogenesis of endometriosis. The microenvironment of FF is complicated in patients with ovarian endometriosis diagnosed by laparoscopy^13,15^. Recent studies have indicated that the metabolomics profiling of FF in endometriosis patients can be employed to assess the possibility of integrating data acquired by different approaches^13,15^. Metabolomics profile has been recommended as a tool which can discriminate between oocytes and embryos with the developmental potential to the blastocysts as well as implantation ability. The utilization rate of amino acid in bovine oocytes has shown to be a good predictive factor of oocyte developmental competence to experience fertilization and early cleavage^12,13^. A variety of studies have indicated that the ultra-long term protocol can improve the quality of oocytes and embryos in patients with moderate to severe endometriosis^16^. The ultra-long term protocol is a common protocol used for the ovulation induction in patients with severe endometriosis. The PPOS protocol is an original protocol developed in our center for ovulation induction, and the goal of this protocol is to inhibit the premature ovulation in the ovulation induction cycle by using progesterone. Our previous study indicated the PPOS protocol in severe endometriosis patients achieves similar oocyte and embryo outcomes to the ultra-long protocol^17^. Studies have confirmed that progesterone can improve the inflammatory status of patients with endometriosis^8^. Thus, we speculate that progesterone in the PPOS protocol may improve the inflammatory status during follicular development in severe endometriosis patients, which is helpful for the improvement of oocyte and embryo quality, finally achieving similar clinical outcomes to ultra-long term protocol. In clinical studies of our group, the pregnancy outcomes of in vitro fertilization-embryo transfer (IVF-ET) after the application of PPOS protocol were explored in the endometriosis patients^18,19^. For these patients, the improvement of inflammatory environment in the follicular development of endometriosis patients is helpful for the improvement of oocyte quality and embryo outcomes. Metabonomic studies have indicated that the energy metabolism and inflammatory metabolism change in the FF of endometriosis patients^15^. However, no metabolomic studies have been undertaken in OE women who underwent the progestin-primed ovarian stimulation (PPOS) protocol. In the present prospective study, metabonomics was employed to compare the metabolites in the FF of endometriosis patients who received IVF-ET with two different ovulation induction protocols (PPOS protocol *vs* ultra-long term protocol). Our findings may provide theoretic evidence on the application of metabonomics in the evaluation of ovulation induction protocol for IVF-ET in the endometriosis patients, which may be helpful for the development of treatment to improve the outcomes of endometriosis patients after IVF-ET.

## Materials and methods

This was a prospective, non-randomized study. Forty infertile women undergoing the first in vitro fertilization /intracytoplasmic sperm injection (IVF/ICSI) in Shanghai Ninth People’s Hospital were consecutively recruited between March 2017 to September 2017. These women were divided into 2 groups depending on the protocols used: 20 women received treatment with the PPOS protocol and 20 women with the ultra-long term protocol (control group). The inclusion criteria were as follows: (1) ovarian endometriomas (> 3 cm) were diagnosed as staged III-IV according to the revised American Fertility Society (AFS) classification^20^; (2) patients were younger than 39 years, but older than 20 years; (3) the baseline follicle-stimulating hormone (FSH) was lower than 10 IU/ml and the antral follicles count (AFC) was greater than or equal to 5; (4) the body mass index (BMI) was in normal range; (5) the menstrual cycle (MC) was regular (27–35 days). Women with male factor infertility, uterine or tubal infertility, or unexplained infertility, polycystic ovary syndrome, premature ovarian failure, hydrosalpinx or adenomyosis were excluded from this study. Of these patients, FF was collected from 20 patients in each group and processed for metabolitics analysis. The study was approved by the Ethics Review Committee of the Ninth People’s Hospital of Shanghai, China ([2014]109). This study has been performed in accordance with the Declaration of Helsinki. Informed consent was obtained from all patients before study.

### Protocols

The MPA + hMG protocol used during IVF has been reported in detail elsewhere^10,11^. HCG (2000 IU) (Lizhu Pharmaceutical Trading Co, Zhuhai, China) and Decapeptyl (0.1 mg) (Ferring International Center SA, Germany) were used at the last stage of oocyte maturation^10^. Viable embryos were cryopreserved. The one-month ultra-long GnRHa scheme was used in the control group. The ultra-long triptorelin at 3.75 mg were administered in patients beginning on MC day 2, and day 5 or 6 weeks later, and hMG was administered at 225 IU once daily. In the GnRHa protocol, hCG (5000 IU) was used at the final stage of oocyte maturation. In the ultra-long term protocol group, fresh embryo transfer was the first choice except for the patients who had to undergo frozen embryo transfer due to some reasons. If the fresh embryo transfer (ET) was not prepared due to high risk of ovarian hyperstimulation syndrome (OHSS), elevated progesterone (P) on the triggering day, unqualified endometrium, or other personal reasons, all good-quality embryos were frozen, and frozen-thawed embryo transfer (FET) was done later.

### Sample preparation and mass spectrometry and liquid chromatography

Under the guidance of vaginal ultrasonography, 2–3 follicles with a diameter of about 18 mm were selected for puncturing, and the FF without obvious blood was used in the following analysis.

The FF was thawed at 4 °C. Then, 200 µL of FF was transferred into a 1.5 mL EP tube, following by addition of 800 µL of methanol. After vertexing for 60 s, the fluid was centrifuged at 12,000 rpm for 10 min at 4 °C. The supernatant was collected and transferred into a 1.5 ml EP tube, followed by drying in the vacuum. The sediment was dissolved with 300 µL of 80% methanol, and the solution was filtered through a filter with the pore size of 0.22 µm. The resultant filtrate was harvested. Then, 20 µL of the filtrate from each sample was mixed into the quality control (QC) sample which was used to adjust the deviation in the assay of mixed sample and the system error. The remaining samples were subjected to LC–MS.

The sample preparation for the metabolomic analysis was based on sample-preparation mean by LC–MS^21^. The specific courses were previously reported^22^. The pooled QC and blank (pure acetonitrile) sample were injected to run for assessing the stability and repeatability. On the basis of QC, quality assurance (QA) was carried out to delete the features with poor repeatability in the QC samples, and higher quality data set was obtained to facilitate the detection of biomarkers. In the QC samples, the proportion of characteristic peaks with RSD < 30% reached 70%, which indicated that the data quality was repeatability. Interleukin-1β (IL-1β), tumor necrosis factor-α (TNF-α) and RANTES concentrations in FF were detected by enzyme linked immunosorbent assay (ELISA; BOSTER, Biological technology Ltd, USA) according to the manufacturer’s instructions. The standard curve was made according to the concentration and optical density (OD) of the standard substance, and then the sample concentration was calculated according to the standard curve. Chromatographic separation was completed in an Acquity LC system. The specific operation process has been described in detail in previous articles^23^.

### Statistical analysis and processing of data

The data were collated by the pheat map package in R version 3.3.2 (Auckland, New Zealand), and the samples and metabolites were bi-directionally clustered. The metabolites were characterized by comparisons with reference standards or MS/MS fragment information obtained from, Human Metabolome Database (HMDB) (http://www.hmdb.ca), Metlin (http://metlin.scripps.edu), LipidMaps (http://www.lipidmaps.org) and mzclound (https://www.mzcloud.org) database. The confirmed metabolites were related to biochemical pathways in KEGG (http://www.kegg.jp/pathway). Metabolic pathway topological analysis and enrichment were implemented using the MetPA database (www.metabo analyst.ca) to evaluate metabolic pathways related to different metabolites^24^. Pearson correlation coefficient was used to estimate the correlation between metabolites^25^. P ≤ 0.05 + variable importance in projection (VIP) ≥ 1 was considered statistically significant indifferences metabolites^26^. Orthogonal Projections to Latent Structures Discriminant Analysis (OPLS-DA) can effectively reduce the complexity of the model and enhance the interpretation ability of the model without reducing the prediction ability of the model, which maximizes the intergroup differences. OPLS-DA uses orthogonal signal correction technology to decompose the X matrix information into Y-relevant and Y-irrelevant information, and then the information that is irrelevant to classification is filtered out. The relevant information is mainly concentrated in the first prediction component. Generally, the Variable Importance for the Projection (VIP) is used to explain the importance of X dataset and relevant Y dataset. The sum of squares of all VIP values are equal to the total number of variables in the model. Thus, its mean is 1. The VIP of larger than 1 is indicative of important role of this variable. Thus, this is usually used for the screening of potential biomarkers.

## Results

### Clinical characteristics of patients

The clinical features of 40 women are displayed in Table 1. No obvious differences were found in the age, infertility duration, baseline levels of hormones, AFC and BMI between 2 groups. Furthermore, the MPA + HMG protocol had a lower stimulation dose of hMG, and the duration of stimulation was significantly different between two groups (P < 0.05). There were no marked differences in the numbers of oocytes retrieved, mature embryos and high quality embryo (P > 0.05).

**Table 1:** Clinical characteristics of patients in two groups.

Characteristics	MPA + HMG group (n = 20)	Ultra-long group (n = 20)	P
Number of cycles	20	20	
Age, years	32.80 ± 3.43	32.46 ± 3.48	0.391
Antral follicle count, n	10.35 ± 3.92	10.57 ± 4.66	0.675
AMH (ng/mL)	3.26 ± 1.43	3.05 ± 1.16	0.562
Hormone levels on day 3			
FSH, IU/L	6.11 ± 1.46	5.74 ± 1.93	0.089
LH, IU/L	3.41 ± 1.39	3.28 ± 1.54	0.061
E2, pg/mL	40.10 ± 16.65	38.90 ± 17.29	0.588
P, ng/mL	0.29 ± 0.12	0.29 ± 0.14	0.595
BMI (kg/m^2^)	21.61 ± 2.15	21.31 ± 2.68	0.231
No. of oocytes punctured	12.68 ± 7.11	13.55 ± 7.49	0.301
hMG dose (IU)	1882.50 ± 388.77	2456.50 ± 560.17*	< 0.001
hMG duration, days	8.72 ± 1.48	11.31 ± 2.26*	< 0.001
MII oocytes (n)	7.77 ± 5.23	8.23 ± 4.93	0.434
Fertilized oocytes (n)	6.73 ± 5.00	6.92 ± 4.35	0.722
High-quality embryos (n)	2.69 ± 2.43	2.43 ± 2.44	0.368

*MPA* medroxyprogesterone acetate, *HMG* human menopausal gonadotropin, *P* MPA + HMG group vs. Ultra-long group.

*P < 0.05 MPA + HMG group vs. Ultra-long group.

### Metabolomics analysis

#### OPLS-DA predicted metabolic profiles

**Figure 1:**
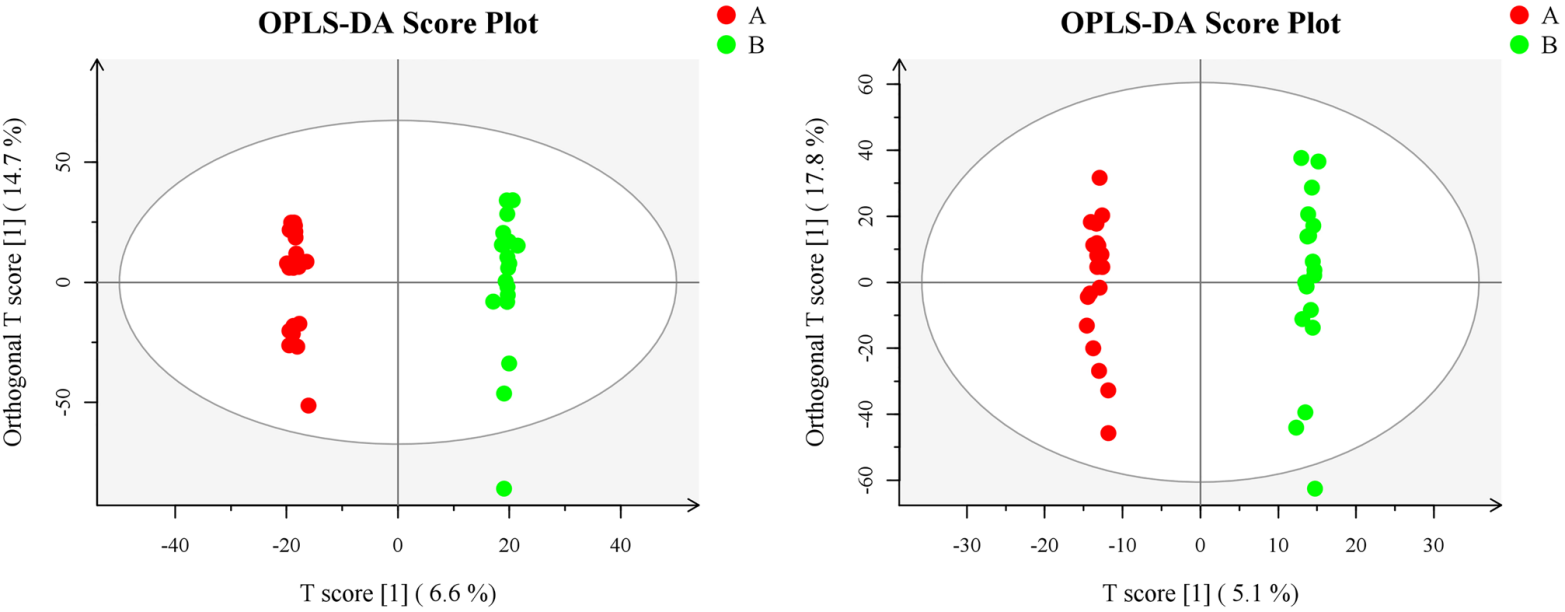
OPLS-DA replacement test chart- positive/negative ion mode, OPLS-DA analysis of the metabolism differences between the two groups.

When redundant peaks were eliminated, LC–MS was applied to test 1158 differential metabolites from each FF sample. In view of the profiled metabolites, OPLS-DA was performed to distinguish PPOS protocol from controls in OE patients. The model exhibited gratifying results in distinction of two groups with one predictive component and one orthogonal component (R2Xcum = 0.306, R2Ycum = 0.998, and Q2cum = 0.886) (Fig. 1). These results revealed that the variation of FF metabolomic profile could be applied to separate two different protocols in OE patients.

#### Biomarker identification and diagnosis evaluation

**Figure 2:**
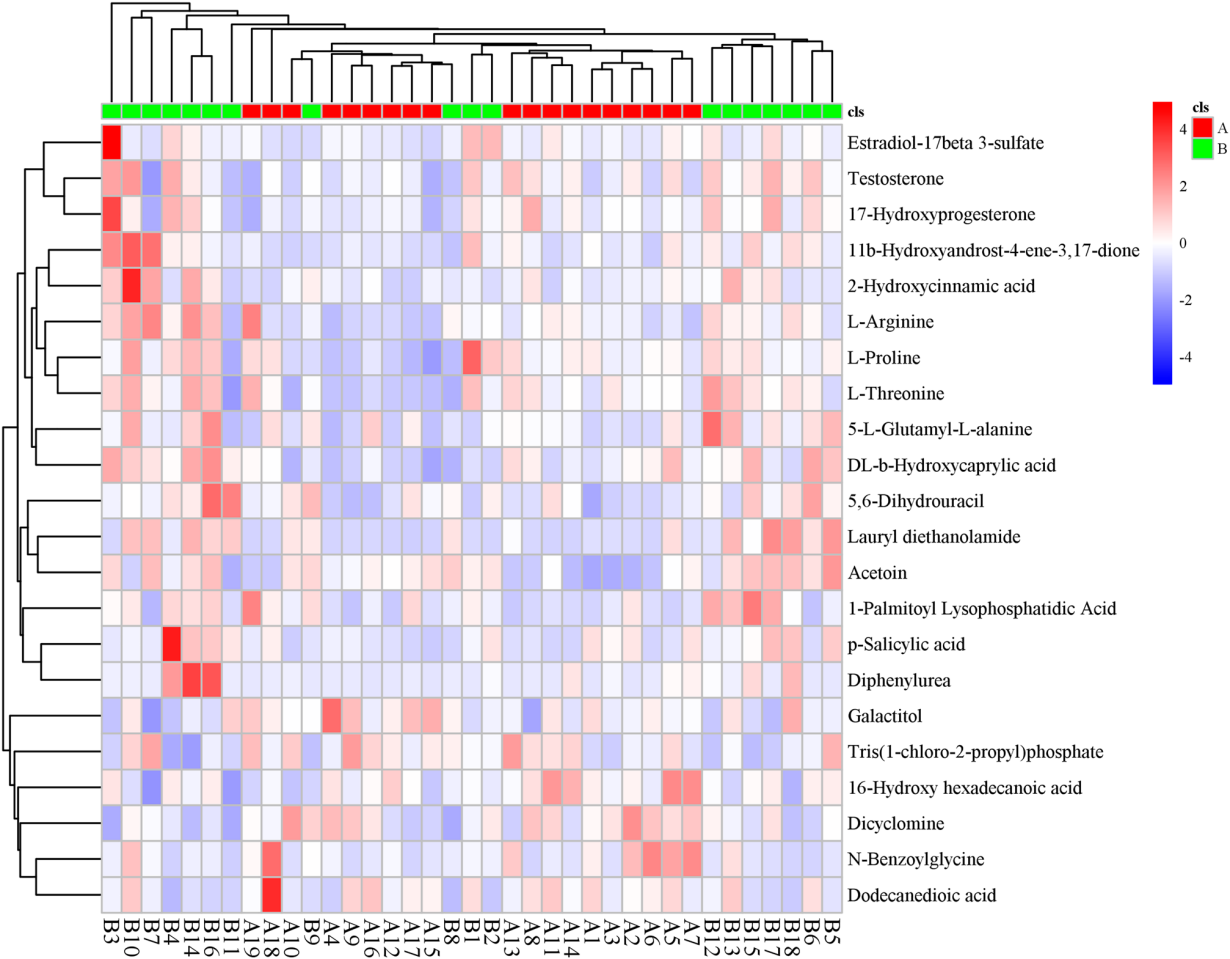
Differential metabolite heat map, significantly changed metabolites between the two groups.

**Figure 3:**
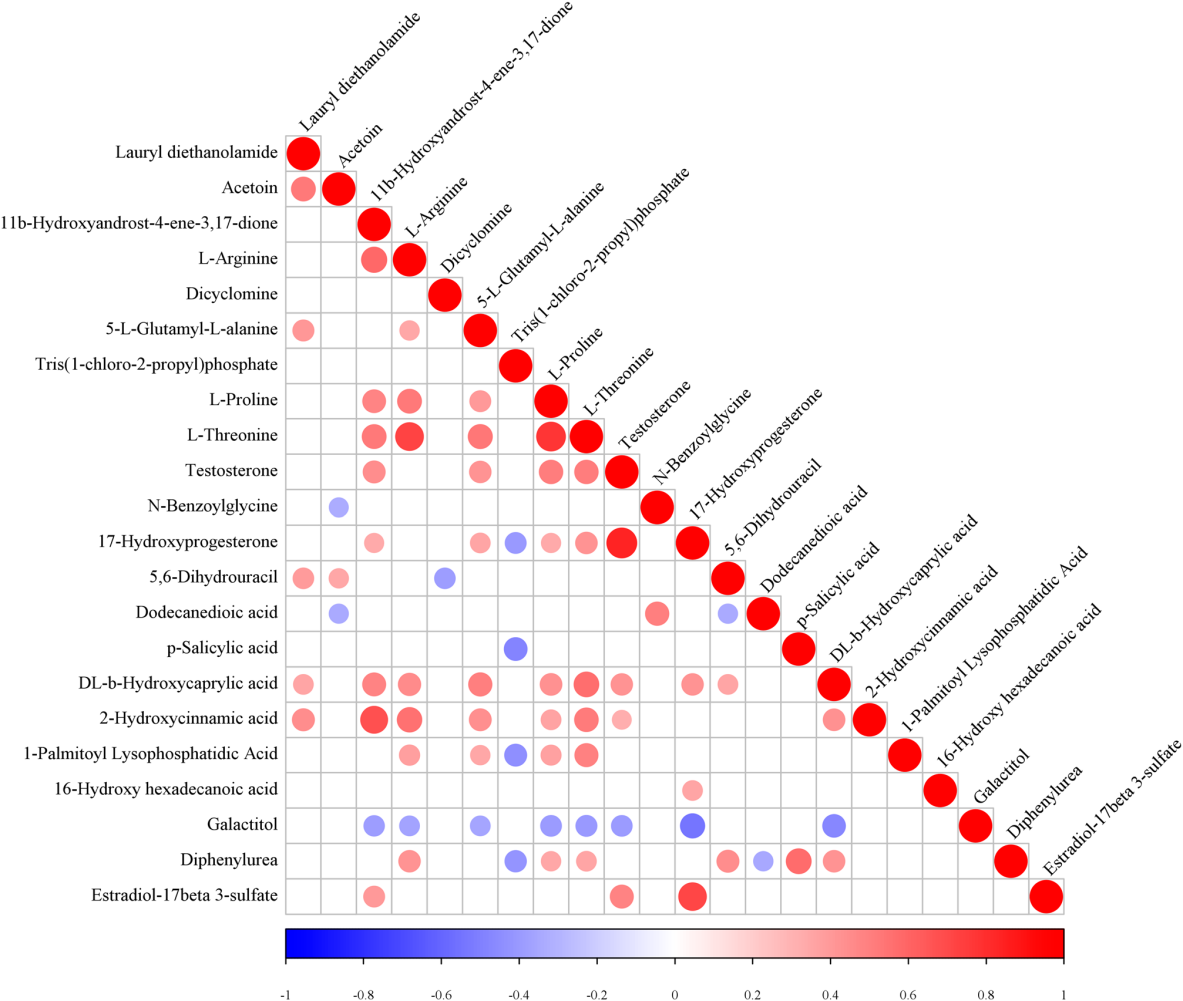
Correlation analysis of changed metabolites. Dark red represents a significant positive correlation. Dark blue represents a significant negative correlation. White represents no correlation.

Variables that were helpful for the distinction of two different protocols in OE patients were considered as potential biomarkers. In Table 2, the analytes that were displayed differentially (nonparametric Wilcoxon–Mann–Whitney, P < 0.005) between two groups were incorporated into the biomarker set. A total of 1158 differential metabolites were established and MS/MS fragments were further confirmed with commercial standards. As described in Table 2, the levels of proline, arginine, and threonine, p-Salicylic acid, glycine, Testosterone, 11b-Hydroxyandrost-4-ene-3,17-dione and 17-Hydroxyprogesteronelevels were significantly different between two groups. Agglomerate hierarchical clustering was used to show the levels of different metabolites in two groups (Fig. 2). The data set was scaled through the heat map package in R (v3.3.2). The differential metabolite correlation analysis was used to study the consistency of the change trend between 19 metabolites. Metabolite correlations often display the synergy of changes among metabolites (Fig. 3).

**Table 2:** Basic characteristics of significantly different metabolites between two groups (1158)^53–55^.

	A vsB VIP	Fold change	mz	rt	Log2 (FC)	P value	FDR
Testosterone	1.307	1.343	289.2155814	855.408	0.425	0.043	0.226
5,6-Dihydrouracil	2.335	1.342	112.9844529	665.39	0.424	0.001	0.051
l-Proline	1.363	1.272	116.0707	100.347	0.348	0.034	0.201
l-Threonine	1.339	1.247	120.0655	92.08885	0.318	0.037	0.211
DL-b-Hydroxycaprylic acid	1.743	1.233	159.1017	477.836	0.302	0.016	0.217
2-Hydroxycinnamic acid	1.734	1.214	163.0391	411.129	0.280	0.017	0.222
Tris(1-chloro-2-propyl)phosphate	1.450	0.985	327.0075	852.667	− 0.022	0.023	0.167
Galactitol	1.583	0.940	180.9722	90.31435	− 0.089	0.031	0.286
Dicyclomine	1.771	0.846	310.271	819.09	− 0.241	0.005	0.063
16-Hydroxy hexadecanoic acid	1.606	0.812	271.2278	892.536	− 0.301	0.028	0.275
Diphenylurea	1.531	3.860	211.0871	797.557	1.949	0.037	0.309
Lauryl diethanolamide	2.489	3.613	288.2526	876.602	1.853	0.000	0.002
Estradiol-17beta 3-sulfate	1.454	1.869	351.127	617.704	0.902	0.048	0.347
11b-Hydroxyandrost-4-ene-3,17-dione	1.843	1.714	303.1948	757.4915	0.777	0.003	0.049
17-Hydroxyprogesterone	1.282	1.426	331.2261	759.185	0.512	0.047	0.237
p-Salicylic acid	1.758	1.389	137.0234	101.6775	0.474	0.015	0.215
Acetoin	2.164	1.384	89.0601	135.382	0.469	0.000	0.011
l-Arginine	1.776	1.381	175.1187	88.02605	0.466	0.005	0.062
Dodecanedioic acid	1.804	0.677	229.1441	541.526	− 0.562	0.013	0.202

*_VIP VIP value, *log2 (FC)* log2 value of fold change, *m/z* mass to charge ratio, *rt* retention time, *FDR* P value adjusted value.

The receiver operating characteristic (ROC) curve was further used to evaluate the identified biomarkers. As displayed in Table 3, 17 metabolites related to PPOS protocol, the area under the ROC curve (AUC) of 17 biomarkers ranged from 0.708 to 0.887, demonstrating that they were moderately predictive markers. Table 3 shows the metabolites with AUC > 0.7.

**Table 3:** Metabolites with AUC > 0.7

	AUC	CI1	CI2	Specificity	Sensitivity	Threshold
Lauryl diethanolamide	0.887	0.686	0.963	0.842	0.833	1.03 × 10^6^
Acetoin	0.86	0.701	0.958	0.842	0.778	1.31 × 10^8^
l-Arginine	0.803	0.687	0.915	0.789	0.833	5.10 × 10^7^
5,6-Dihydrouracil	0.791	0.647	0.903	0.842	0.722	1.83 × 10^8^
Dodecanedioic acid	0.778	0.655	0.904	0.789	0.722	4.52 × 10^5^
p-Salicylic acid	0.768	0.554	0.902	0.737	0.722	7.33 × 10^5^
11b-Hydroxyandrost-4-ene-3,17-dione	0.766	0.588	0.895	0.789	0.722	4.30 × 10^5^
Dicyclomine	0.76	0.587	0.887	0.684	0.722	2.37 × 10^7^
1-Palmitoyl Lysophosphatidic acid	0.74	0.586	0.899	0.789	0.722	7.63 × 10^6^
Icaridin	0.716	0.592	0.839	0.842	0.667	8.92 × 10^6^
17-Hydroxyprogesterone	0.715	0.537	0.88	0.684	0.778	3.22 × 10^7^
Tris(1-chloro-2-propyl)phosphate	0.712	0.522	0.835	0.579	0.833	6.31 × 10^7^
Diphenylurea	0.711	0.559	0.83	0.737	0.722	1.91 × 10^5^
Galactitol	0.709	0.533	0.883	0.789	0.667	4.81 × 10^7^
L-Proline	0.709	0.517	0.839	0.737	0.556	1.30 × 10^9^
DL-b-Hydroxycaprylic acid	0.708	0.545	0.87	0.684	0.667	1.69 × 10^6^
Testosterone	0.708	0.489	0.844	0.632	0.722	7.68 × 10^6^

*AUC* area under ROC curve, *CI1* 95% CI lower limit, *CI2* 95% CI upper limit.

### Efficacy of PPOS on the metabolic pathway of OE

**Figure 4:**
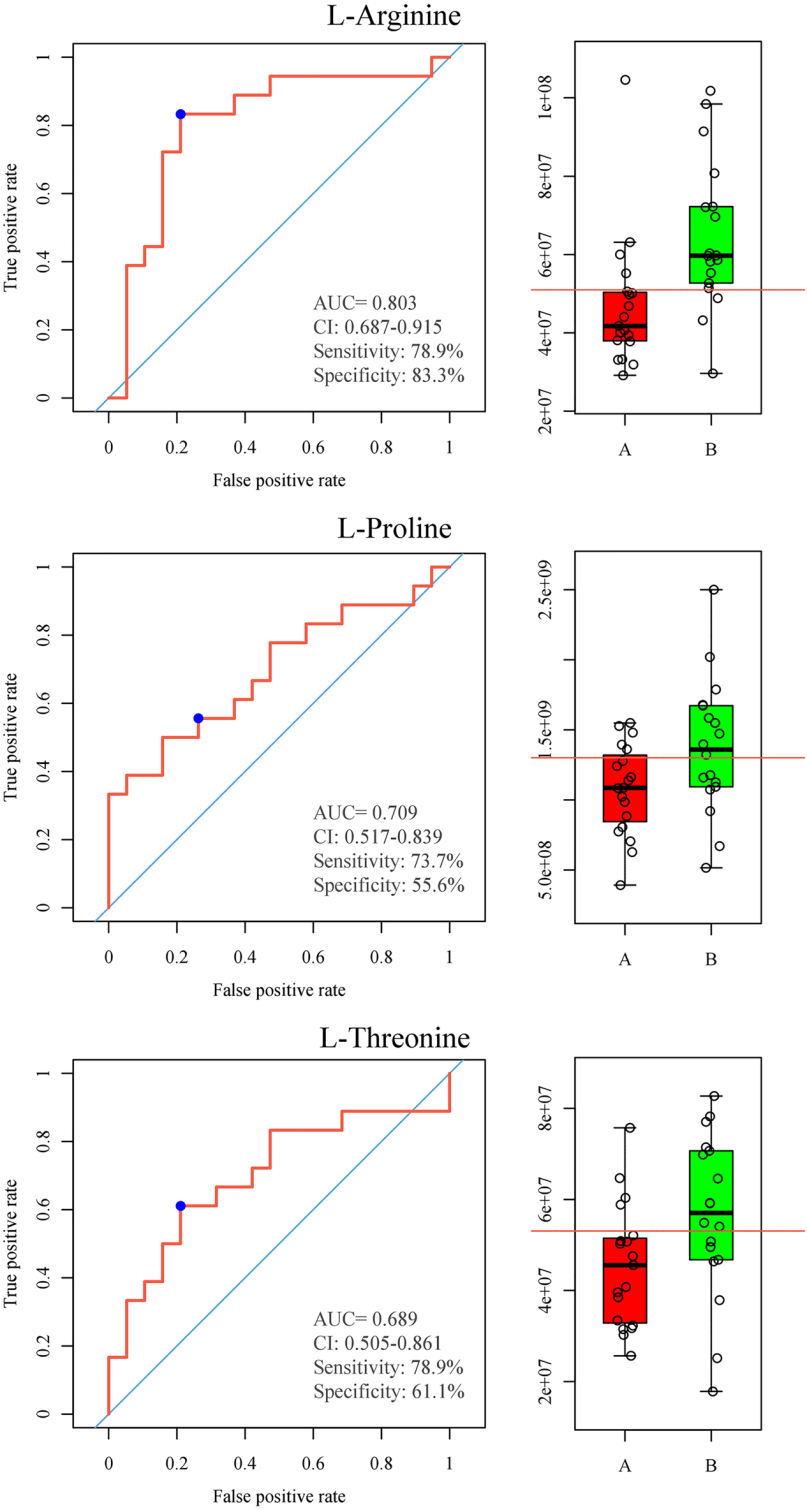
Arginine-box plot and Arginine-ROC curve in two groups, Proline-box plot and Proline-ROC curve in two groups, Threonine-box plot and Threonine-ROC curve in two groups.

Multivariate analysis was performed on the differential metabolites to test the efficacy of PPOS on relevant metabolite pathways. The more discrete the point, the greater the disturbance was. The influences of PPOS on 17 metabolic pathways of FF were analyzed, and results showed the most affected pathways were phenylalanine metabolism pathway, which involves glycine, salicylic acid and cinnamic acid, and the aminoacyl-tRNA biosynthesis pathway, arginine and proline metabolism pathway, which involves proline (AUC: 0.709), arginine (AUC: 0.803) and threonine (AUC: 0.689) (Fig. 4; Table 4).

**Table 4:** Related metabolic pathways involved in this experiment.

	Total	Hits	Rawp	Log (p)	Ajust	FDR	Impact	Compounds	KEGG path
Phenylalanine metabolism	45	3	0.004096	5.4977	0.32768	0.21127	0.04177	C01586; C01772; C00156	hsa00360
Steroid hormone biosynthesis	99	4	0.0052817	5.2435	0.41725	0.21127	0.12129	C01176; C05284; C00535; C08357	hsa00140
Aminoacyl-tRNA biosynthesis	75	3	0.016933	4.0785	1	0.45154	0	C00062; C00188; C00148	hsa00970
D-Arginine and D-ornithine metabolism	8	1	0.058366	2.841	1	1	0	C00062	hsa00472
Arginine and proline metabolism	77	2	0.11115	2.1969	1	1	0.22863	C00062; C00148	hsa00330
Pantothenate and CoA biosynthesis	27	1	0.18436	1.6909	1	1	0.02002	C00429	hsa00770
Valine, leucine and isoleucine biosynthesis	27	1	0.18436	1.6909	1	1	0	C00188	hsa00290
beta-Alanine metabolism	28	1	0.19052	1.658	1	1	0.02328	C00429	hsa00410
Ubiquinone and other terpenoid-quinone biosynthesis	36	1	0.23831	1.4342	1	1	0.03049	C00156	hsa00130
Glutathione metabolism	38	1	0.24984	1.387	1	1	0.02094	C03740	hsa00480
Galactose metabolism	41	1	0.26681	1.3212	1	1	0.08543	C01697	hsa00052
Glycine, serine and threonine metabolism	48	1	0.30503	1.1874	1	1	0.09661	C00188	hsa00260
Pyrimidine metabolism	60	1	0.36619	1.0046	1	1	0.01492	C00429	hsa00240
Porphyrin and chlorophyll metabolism	104	1	0.54973	0.59832	1	1	0	C00188	hsa00860

*Total* the Total number of metabolites in the target metabolic pathway, *Hits* number of different metabolites in the target metabolic pathway, *Raw P* p value for hypergeometric distribution test, *− log(p)* take a negative value of the natural log of p, *Holm adjust* p value after Holm false positive correction, *FDR* false positive corrected value, *Impact* the impact value of metabolic pathways.

### Inflammatory factors in two groups

The levels of IL-1β, RANTES and TNF-α in the FF of PPOS protocol group were considerably lower than in the ultra-long protocol group (IL-1β: 32.25^23,37^ ng/mL *vs* 35.04^30,49^ ng/mL; RANTES: 231.81 ± 21.41 ng/mL *vs* 258.42 ± 16.26 ng/mL; TNF-α: 48.88 ± 5.26 ng/mL *vs* 58.01 ± 7.23 ng/mL; P < 0.05).

## Discussion

In the present study, LC–MS was performed to explore the metabolomics profile of FF in patients undergoing IVF/ICSI with PPOS or ultra-long term protocol. Three differentially expressed amino acids (proline, arginine, and threonine) were identified to be associated with the PPOS protocol that achieved high-quality oocytes and satisfactory pregnancy outcomes^17^. The levels of IL-1β, RANTES and TNF-α in the FF of PPOS protocol group were remarkably lower than in the ultra-long protocol group. Metabolomics analysis of the FF has allowed the identification of biomarkers and metabolic pathways related to several pathologies which affect oocyte quality. In the present study, the metabolic pathways related to the oocyte quality included phenylalanine metabolic pathway, synthesis of aminoacyl proline and arginine metabolism.

The administration of GnRH agonists before IVF/ICSI may advance the pregnancy rate. The 2006 Cochrane guideline proposes the use of GnRH analogs for more than 2 months before IVF cycle to “inhibit the inflammatory state”, thereby improving the pregnancy rate^16^. It has been confirmed that endometriosis is detrimental to the ovaries. Toxic factors (such as reactive oxygen species [ROS], free iron and inflammatory molecules) from an endometrioma may cause adverse events such as increased oxidative stress, reduced follicular maturation and impaired fertilization^27,28^. A 3-month ultra-long GnRHa creates a more favorable environment for oocyte maturation, with better oocyte and embryo quality as well as fertilization rate. The effect of ultra-long term protocol on the oocyte and embryo quality is still controversial. Some studies indicate that the ultra-long term protocol is able to improve the oocyte and embryo quality, but others fail to reveal the improvement of oocyte and embryo quality after the ultra-long term protocol^29^. Whether this vicious cycle of damage can be ameliorated by MPA treatment during IVF/ICSI is still unclear. MPA is clinically useful for the treatment of pain and may improve the overall comforts in most women with endometriosis^30,31^. There is still controversy on the therapeutic efficacy of MPA, such as danazol, stems from the pituitary inhibition and atrophy of endometriotic lesions^32^. Another possible attribute is that MPA alleviates inflammation at the site of endometriotic implants. Prolonged treatment (8 days) with MPA decreases the luciferase activity by 36% and reduces the RANTES protein expression by 50%. Nevertheless, short treatment (2 or 4 d) with MPA has no significant effect. Our previous study also showed that 8-d MPA increased progesterone receptor (PR) expression^8^. General metabolite profiling has been supposed to be a tool which can distinguish between pronucleate zygotes and embryos with the developmental potential into the blastocysts and implantation^12^. In the present study, no significant differences were observed in the number of mature oocytes, number of high-quality embryo and fertilization, and pregnancy rate between two groups. Although the PPOS protocol did not significantly elevate the pregnancy rate, it at least achieved the pregnancy outcome similar to those after ultra-long term protocol^17^, and the inflammatory cytokines reduced significantly, suggesting the improvement of microenvironment of oocyte development. These findings support that oocyte metabolism reflects oocyte quality. Thus, more studies are needed to investigate the association of functional metabolites with the quality of human oocytes having embryo developmental competence. Our study was to explore the differential metabolites during the COH between endometriosis patients with PPOS protocol and those with ultra-long term protocol. Some studies shown that individual bovine MII oocytes with the capacity to support zygote cleavage and blastocyst development in vitro following IVF display a different preference for amino acids compared with oocytes which failed to fertilize or undergo embryo cleavage^33^. The study also demonstrated, that amino acid depletion/appearance by human oocytes is a function of patient age, and gonadotrophin preparation and dose used for COS^12^. These findings indicate that metabolite profiling is useful to identify the ‘nutritional fingerprint’ related to the oocyte quality in endometriosis patients, which may be used to enhance the efficacy of ART^33^.

The signalling pathways that promote the resumption and progression of meiosis of bovine oocytes after the luteinizing hormone (LH) peak are not fully understood^34^. One of the factors involved in the oocyte maturation that have been investigated is nitric oxide (NO). NO is produced by the nitric oxide synthase (NOS), which catalyzes the production of l-citrulline and NO from l-arginine (l-Arg). The utilization rate of L-Arg for NO synthesis in the medium is one of the keys in the in vitro synthesis of NO^35^.

It has been confirmed that some key enzymes and metabolites in the arginine metabolism pathway play important roles in the development of follicles^36^. They can directly affect the follicles, or indirectly improve the development of follicles by promoting the proliferation of granulosa cells. Therefore, the arginine level after the use of PPOS regimen in patients with endometriosis significantly increased, which may be a key factor to improve the developmental microenvironment of follicles and regulate the function of granulosa cells.

At the same time, FF has its own antioxidant system, such as superoxide dismutase (SOD)^37–39^. The antioxidative activity of FF has an adverse effect on the oocyte and embryo developmental potential as well as pregnancy outcome^40^. The increased ROS are the principal consideration which influences mammalian embryo development in vitro^41,42^. The roles of dietary supplementation with arginine in the regulation of inflammation, antioxidation, and mRNA expression of antioxidation-related molecules in the spleen have been confirmed in rats under oxidative stress^43^. Studies have shown that Arg facilitates the protein synthesis, improves the reproductive performance, and facilitates the intestinal cell migration^44,45^. These indicate that Arg may influence the antioxidative defense in the spleen. The antioxidative capacity has a correlation with the inflammatory response^46^, which is regulated by some inflammatory cytokines. In pigs, Arg is able to decrease the transcription of TNF-αand IL-6 in the jejunum challenged by lipopolysaccharide^47^. Moreover, Arg intake may increase the expression of transforming growth factor-β (TGF-β) and IL-10 in carps, which is partially ascribed to the upregulated mTOR expression^48^. Further investigations indicate that this may be affected by the transcription factor nuclear factor κB (NF-κB), which may induce the resistance to programmed cell death by up-regulating the expression of anti-apoptotic genes^49^. These findings indicate that Arg may affect the pro-inflammatory and anti-inflammatory cytokines in animals^50^. In our study, the levels of IL-1β, RANTES and TNF-α in the FF of PPOS protocol group remarkably reduced as compared to the ultra-long term protocol group. LC–MS has been used to compare the metabolites of FF between non-endometriosis and endometriosis samples^15^. Results showed β-hydroxybutyric acid and glutamine significantly increased, whereas tryptophan markedly decreased in the endometriosis patients. Karaer et al.^51^ conducted metabonomic analysis of FF, and results showed that lactic acid, β-glucose, pyruvate and valine significantly increased in the FF of endometriosis women when compared with the controls, which was related to the inhibition of follicular development in these women. In patients with repeated fertilization failure, valine in the FF increased, which may be related to infertility in these patients. Marianna et al.^52^ found the levels of alanine, proline, aspartic acid, glucose, valine, leucine, lysine, choline and phosphocholine in the FF decreased, while those of lactic acid, phospholipid and other lipids increased in the FF of endometriosis women. Excessively high or low levels of these metabolites may exert adverse effects on the follicular development. Increased metabolites may induce oxidative stress in the FF, affecting the cytoplasm and nucleus of oocytes. Valine, leucine and lysine are essential amino acids, leucine and lysine are ketogenic amino acids, and lysine is a branched chain amino acid. They are involved in the synthesis of ATP and can be metabolized into ketone bodies. The reduction of these amino acids is closely related to the abnormal energy metabolism in the FF. Proline, arginine, threonine, and glycine significantly increased in women with PPOS protocol as compared to controls. These amino acids are also related to the energy metabolism and inflammation, and may be involved in the ovulation induction with PPOS protocol.

No study has been conducted to explore the antioxidative effect of L-Arg in the FF of patients with endometriosis. The progression of endometriosis is estrogen dependent, while progesterone can suppress the effect of estrogen. Although the estrogen level in the PPOS protocol group was significantly higher than in the ultra-long protocol group, the levels of inflammatory factors significantly reduced. The relationship between l-Arg level and expression of antioxidant enzymes is still unclear. However, above studies showed that the arginine content significantly increased in patients with endometriosis receiving PPOS protocol, and arginine might promote the development of oocytes and embryos by improving the inflammation and immune status of patients with endometriosis through important signaling pathways.

The phenylalanine metabolism is interesting. In the plasma, phenylalanine may be metabolized into tyrosine, mainly in the liver. Tyrosine is closely related to some hormones and neurotransmitters. Thus, it would be a kind of “environmental estrogen” effect where the action of estrogens is modulated by other phenolic compounds.

There were still limitations in the present study. In our study, ovarian endometriosis patients were included, and they might have deep endometriosis. Endometriosis is a heterogeneous disease, and its influence on the FF may be dependent on the severity of endometriosis. In the ultra-long term protocol, the conventional pre-treatment often lasts for 3 months or more, but the time of downregulation is longer, side effects are serious, medical cost is high, the treatment is long, the therapeutic response is poor, and the patient’s compliance is also unacceptable, which may cause early ending of clinical studies. Thus, in our study, the time of downregulation was one cycle. In addition, there was no ideal control group to study the metabolic characteristics of FF in endometriosis patients. The endometriosis patients receiving IVF for tubal infertility will be the suitable controls. In addition, the sample size was small in the present study, which might bias the results of pregnancy outcomes. Thus, the pregnancy outcomes were not displayed in this study. Further studies with large sample size and elegant design are needed to confirm our findings and reveal the mechanism(s) for the alteration of metabolite profiles in the FF.

## Data Availability

All data generated or analysed during this study are included in this published article.
